# Pharmaceutical Market Access: current state of affairs and key challenges – results of the Market Access Launch Excellence Inventory (MALEI)

**DOI:** 10.3402/jmahp.v3.29679

**Published:** 2015-12-21

**Authors:** Marcus A. Koch

**Affiliations:** ^a^ Laboratoire de Santé Publique, Faculté de Médecine, Aix-Marseille Université, Marseille, France

**Keywords:** Market Access Launch Excellence, inventory, benchmarking, life sciences, biopharmaceutical industry, product life cycle, implementation gaps, activity model, early HTA, beyond the pill, talent management

## Abstract

**Objectives:**

To take inventory of the current state of affairs of Market Access Launch Excellence in the life sciences industry. To identify key gaps and challenges for Market Access (MA) and discuss how they can be addressed. To generate a baseline for benchmarking MA launch excellence.

**Methodology:**

An online survey was conducted with pharmaceutical executives primarily working in MA, marketing, or general management. The survey aimed to evaluate MA excellence prerequisites across the product life cycle (rated by importance and level of implementation) and to describe MA activity models in the respective companies. Composite scores were calculated from respondents’ ratings and answers.

**Results:**

Implementation levels of MA excellence prerequisites generally lagged behind their perceived importance. Item importance and the respective level of implementation correlated well, which can be interpreted as proof of the validity of the questionnaire. The following areas were shown to be particularly underimplemented: 1) early integration of MA and health economic considerations in research and development decision making, 2) developing true partnerships with payers, including the development of services ‘beyond the pill’, and 3) consideration of human resource and talent management. The concept of *importance-adjusted implementation levels* as a hybrid parameter was introduced and shown to be a viable tool for benchmarking purposes. More than 70% of respondents indicated that their companies will invest broadly in MA in terms of capital and headcount within the next 3 years.

**Conclusions:**

MA (launch) excellence needs to be further developed in order to close implementation gaps across the entire product life cycle. As MA is a comparatively young pharmaceutical discipline in a complex and dynamic environment, this effort will require strategic focus and dedication. The Market Access Launch Excellence Inventory benchmarking tool may help guide decision makers to prioritize their endeavors.

Launching new products successfully is critical for the life sciences industry. Not only is it important for bringing new reimbursed medicines, diagnostics, and medical technologies quickly to patients in need, it is also vital for companies to recoup their R&D investments and to overcome revenue shortfalls related to maturing products and patent expirations. Launching new products or indications fuels profit growth, which is then used for the advancement of healthcare innovation through continuous investments in R&D and, last but not least, to increase shareholder value and to secure the future existence of a company. Launches are ‘moments of truth’, where all the cross-functional efforts of a company to advance a product through development and commercialization come together into success or failure as measured by market uptake versus the company's expectations. Launches are difficult to get right. An analysis of 210 new molecular entities first launched between 2003 and 2009 showed that 66% delivered sales in their first year of launch below forecast, 8% delivered on or near forecast and only 26% performed better than forecast (1).

When evaluating the pipeline of the top 25 pharmaceutical companies based on 2014 revenue (2), the future looks bright. Companies are faced with a wave of new launches in 2015–2016 and beyond (3). Even after accounting for a likelihood of approval of 83% for late-stage pipeline products (4) there is likely to be an increase of around 66% in product launches in 2015–2016 versus baseline in 2010–2014 (see Fig. 1). This increased launch workload will require pharmaceutical companies to develop distinct launch approaches based on segmentation as well as focusing and building up of launch expert resources (5).

**Fig. 1.**
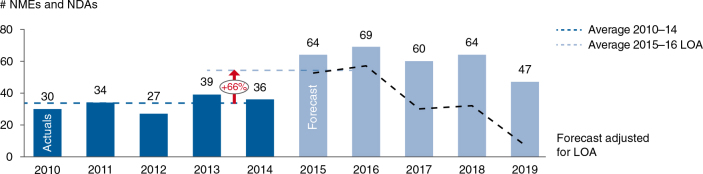
The top 25 pharmaceutical companies’ product launches 2010–2019: ~66% more launches per annum in 2015–2016 versus 2010–2014. NMEs, new molecular entities (including biologics); NDAs, new drug applications (as defined by EvaluatePharma^®^); LOA, likelihood of approval.

Healthcare systems across the world have undergone unprecedented levels of change in part triggered by the global economic crisis in 2008. Their primary aim now is containing health spending growth. Health expenditure accounts for a significant portion of gross domestic product (GDP) (5–11% in EU-25, 16% in the United States, based on 2012 data) (6) and continues to outgrow GDP (see Fig. 2), which poses an enormous challenge for payers in managing their budgets. Pharmaceutical spending accounted for between 14 and 18% of health expenditure in EU-4 and 12% in the United States in 2012 (7) (see Fig. 2) with growth successfully contained since 2008, even disproportionally more than overall health spending growth. This containment is due to numerous health reforms primarily geared towards reducing pharmaceutical expenditure. A total of 116 reforms were implemented or planned in Europe alone from 2010 to 2011 (8). However, payers still believe that drug costs are a major budget driver (9). Affordability appears to be the key issue. Therefore, scrutiny of medicines’ effectiveness and budget impact is likely to stay high. Payers seek to optimize the anticipated health gain of a drug versus budget impact versus the available budget in order to distribute their scarce resources in the most efficient way. Cost containment measures and reimbursement practices that address payers’ uncertainty about a new product's incremental value are continuously evolving and spreading. They are heterogeneous at the country, subnational, and individual account levels. More recently, the Affordable Care Act (ACA) and the emergence of accountable care organizations (ACOs) in the United States, as well as Market Access (MA) agreements tying reimbursement to outcomes as introduced in several legislations in Europe, have unleashed experiments on ‘pay for performance’ (P4P) and various risk-sharing schemes for reimbursement. The aim is to incentivize the provision of healthcare based on health outcomes, in an attempt to switch the funding process, maximize health, and minimize unnecessary services (10). This has triggered a rethink of healthcare towards a systemic, integrated delivery model. Such a fundamental shift will necessitate a collaborative and co-responsible endeavor in partnership between payers, healthcare providers, and the life sciences industry. For companies, this shift will ultimately mean an evolution from a predominantly product-centric business model towards the provision of healthcare solutions and health outcomes.

**Fig. 2.**
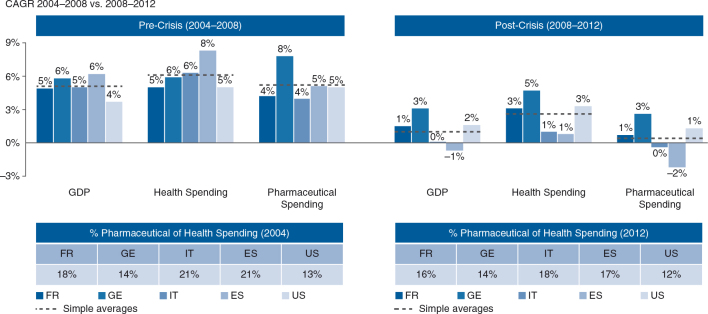
Compound annual growth rates (CAGRs) of gross domestic product (GDP), per capita total health and pharmaceutical expenditures for France, Germany, Italy, Spain, and the United States in 2004–2008 versus 2008–2012. The pharmaceutical industry has contributed disproportionally to cost containment since the 2008 crisis.

The discipline of MA, defined broadly as ‘the integration of pricing and reimbursement, health economics and outcomes research, policy/corporate affairs and patient advocacy’, plays a central role in securing excellent launches on time. While confronted with an increasing launch workload, MA must take into account an extremely volatile reimbursement environment, which it ideally coshapes. MA has a strong spatiotemporal component with points of activation along the product life cycle: roughly pre-, peri-, and postlaunch. Furthermore, MA operates at the intersection of R&D, medical and regulatory affairs, and commercial and involves several entities of a company: the corporate center, regional headquarters, and country affiliates. The author acknowledges that launch excellence is ultimately a cross-functional endeavor. However, given the increasing criticality and importance of MA for launch and company success, a definition of *launch excellence* from a MA perspective is warranted: ‘*MA launch excellence* defines appropriate organization and governance, processes, tools, and skills to ensure the integration of the perspectives of payers and patients at each relevant stage of pharmaceutical development and commercialization, in order to create compelling value propositions for successful launches’.

This study therefore takes an inventory of the current state of affairs of MA from a holistic standpoint and with a focus on launches. It attempts to cover the spatiotemporal aspect of MA and looks at both structural and cultural factors. It provides a snapshot of where the discipline of MA currently stands and where the key challenges and gaps currently are from an industry insider's viewpoint.

## Methodology

### Questionnaire generation

A questionnaire for an online survey, termed *Market Access Launch Excellence Inventory* (MALEI), was created. The questionnaire was divided into three blocks: 1) details of company and respondent; 2) MA activities in the respondent's company entity (only directed to respondents working in either pricing and reimbursement, health economics and outcomes research, policy/corporate affairs, or patient advocacy); 3) the MALEI core assessment, which consisted of three sections: A) organization and governance, B) tools and frameworks, and C) skills and competencies. Within each section, survey respondents were presented with between 6 and 11 MA excellence items and were asked to rate them based on two criteria: *importance* and *level of implementation* within their company. For both criteria, five point rating scales were used with an additional evasive option in case the respondent did not know or had no opinion. The scale of importance was indicated as follows: 1 – unimportant, 2 – of little importance, 3 – moderately important, 4 – important, 5 – very important. The level of implementation was shown as follows: 1 – not at all implemented, 2 – a little implemented, 3 – somewhat implemented, 4 – substantially implemented, 5 – fully implemented. MALEI items were selected based on the author's experience of his work in the biopharmaceutical industry and management consulting, as well as knowledge gained from his recent university studies of European Market Access at Aix-Marseille University (EMAUD). This approach was complemented by discussions with experts and colleagues and a review of current literature. A PubMed search did not yield any relevant articles; however, Google searches delivered several viewpoints and publications from leading consulting companies on various aspects of MA within the life sciences industry, which were implicitly considered (5, 11–15). The MALEI list of excellence prerequisites does not claim to be exhaustive.

### Survey implementation

The survey was conducted online from May to July 2015 using the SurveyMonkey platform (www.surveymonkey.com). A total of 284 executives from life sciences companies were invited to participate in the survey, either via an e-mail from the author and the office of Interpharma, the association of research-based pharmaceutical companies in Switzerland, or via the LinkedIn EMAUD alumni group. Seventy responses were obtained (response rate 25%), of which 61 were (partially) complete (completion rate 87%).

### Analysis

MALEI items were clustered by development and commercialization stage and the three MALEI assessment sections as outlined above. Survey responses were analyzed using Microsoft Excel. Composite rating scores were calculated as simple averages. *Importance-adjusted implementation levels* (IAILs) for all MALEI prerequisites were calculated as the quotient of implementation level and importance rating. Evasive responses (‘no opinion’, ‘do not know’) were excluded from calculations.

## Results

### Survey demographics

**Fig. 3.**
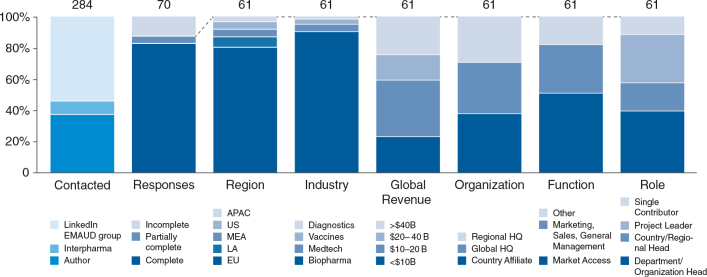
MALEI survey demographics.

Sixtyone (partially) complete responses were considered for analysis (see Fig. 3). Of the respondents, 80% were from Europe, 90% of whom worked for an originator biopharmaceutical company. Responses were almost evenly distributed across country affiliates, regional headquarters, and global headquarters. Fifty percent of survey respondents worked in MA; 30% in marketing, sales, or general management; and 20% in other areas (primarily in medical and regulatory affairs). The proportion of respondents working in leadership roles was 90%.

### MA Launch Excellence Inventory

**Fig. 4.**
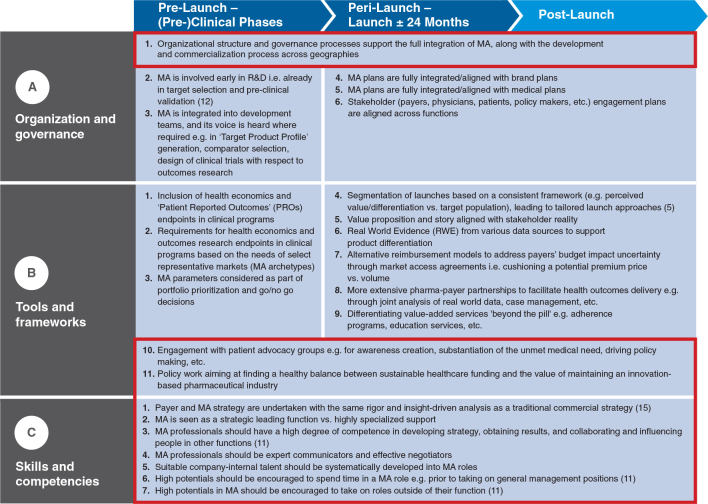
MALEI items as used in the survey, clustered by product life cycle stage and key topics. Life cycle overarching excellence prerequisites are highlighted with red boxes.

**Fig. 5.**
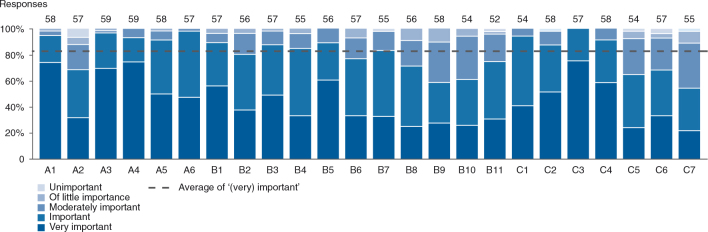
MALEI prerequisites by importance. Items have been rated as ‘(very) important’ by 82% of respondents on average.

**Fig. 6.**
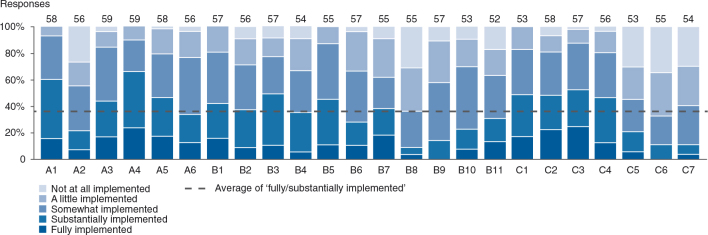
MALEI prerequisites by level of implementation. Items have been rated as ‘fully/substantially implemented’ by 36% of respondents on average. Implementation levels clearly lag behind perceived importance (compare with Fig. 5).

**Fig. 7.**
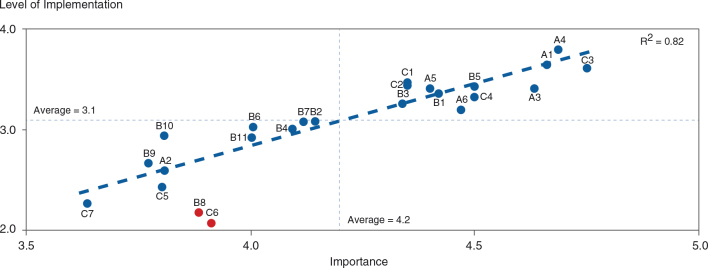
The level of implementation is a function of importance with good correlation (*n*=52–59). Two MALEI items are clearly below the regression line and can be considered as underimplemented versus their perceived importance: B8 (‘more extensive pharma–payer partnerships to facilitate health outcomes delivery’) and C6 (‘high potentials should be encouraged to spend time in a MA role, e.g., prior to taking on general management positions’).

**Fig. 8.**
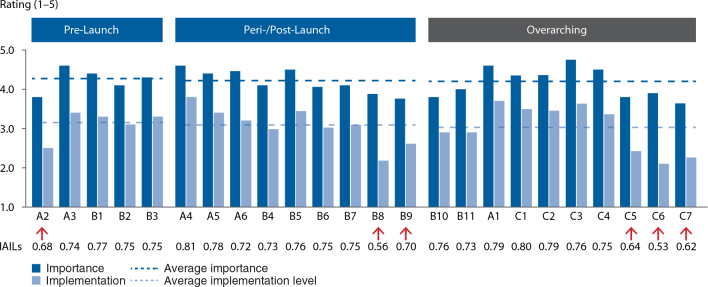
Clustering of MALEI scores by life cycle stage and overarching parameters (*n*=52–59). Each cluster contains MALEI prerequisites that are underimplemented versus cluster average. In addition to the items already identified in the regression analysis (B8 and C6, see Fig. 7), these are as follows: A2 (‘MA is involved early in R&D, i.e., already in target selection and preclinical validation’), B9 (‘differentiating value-added services “beyond the pill”’), C5 (‘suitable company-internal talent should be systematically developed into MA roles’), C7 (‘high potentials in MA should be encouraged to take on roles outside of their function’). MA, Market Access; IAILs, importance-adjusted implementation levels.

An overview of the MALEI excellence prerequisites clustered by product life cycle stage and MALEI assessment section is provided in Fig. 4; 10 of the 24 excellence items were found to span the entire life cycle: A1, B10-11, C1-7. MALEI items were rated as (very) important by 82% of respondents on average, which can be seen as proof of the relevance of the MALEI questionnaire (see Fig. 5). However, implementation of the excellence prerequisites is clearly lagging behind, with an average of only 36% of respondents rating items as fully or substantially implemented (see Fig. 6). The level of implementation has been shown to be a function of importance with a good correlation (*R* ^2^=0.82; see Fig. 7), which can be interpreted as proof of the validity of the questionnaire. In this analysis, two MALEI items can be clearly identified as underimplemented versus their perceived importance: B8 (‘more extensive pharma–payer partnerships to facilitate health outcomes delivery’) and C6 (‘high potentials should be encouraged to spend time in a MA role, e.g., prior to taking on general management positions’). Clustering of MALEI scores by development and commercialization stage and overarching parameters (see Fig. 8) revealed that neither cluster stands out in terms of importance (average rating 4.2–4.3) or level of implementation (average rating 3.0–3.2). Supporting the regression analysis shown in Fig. 7, this analysis allowed for the detection of MALEI items that are underimplemented compared with the respective cluster average. In the prelaunch cluster, A2 (‘MA is involved early in R&D, i.e., already in target selection and preclinical validation’) appears to be underimplemented, as are B8 (already identified, see above) and B9 (‘differentiating value-added services beyond the pill’) in the peri-/postlaunch cluster. In the product life cycle overarching cluster, human resource and talent development shows implementation gaps, with three relatively underimplemented survey items: C5 (‘suitable company-internal talent should be systematically developed into MA roles’), C6 (already identified, see above), and C7 (‘high potentials in MA should be encouraged to take on roles outside of their function’). Furthermore, Fig. 8 highlights IAILs, which normalize the degree of implementation for importance. IAILs can serve for benchmarking purposes (see Discussion and Conclusions).

### Current and future MA activity models

**Fig. 9.**
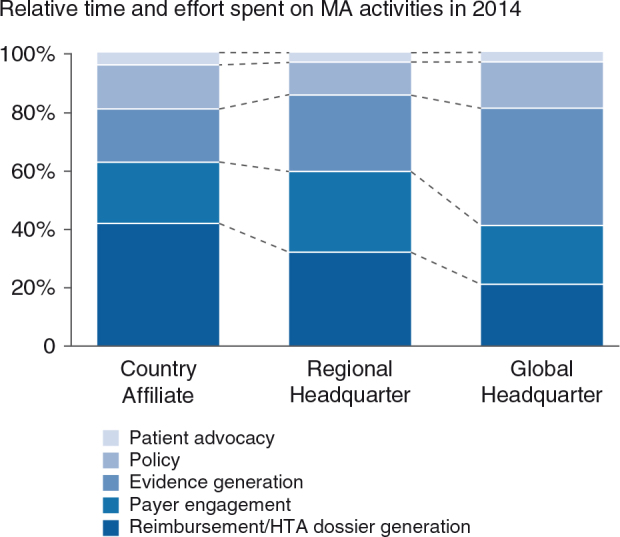
The current MA activity model by organizational entity [*n*(AFF)=10, *n*(RHQ)=9, *n*(GHQ)=7]. AFF, country affiliates; RHQ, regional headquarters; GHQ, global headquarters.

**Fig. 10.**
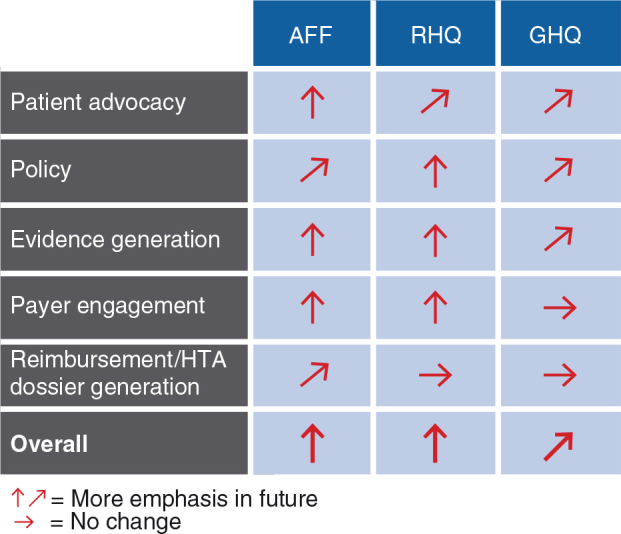
The future MA activity model: virtually all activities to be expanded [*n*(AFF)=10, *n*(RHQ)=9, *n*(GHQ)=7]. AFF, country affiliates; RHQ, regional headquarters; GHQ, global headquarters.

**Fig. 11.**
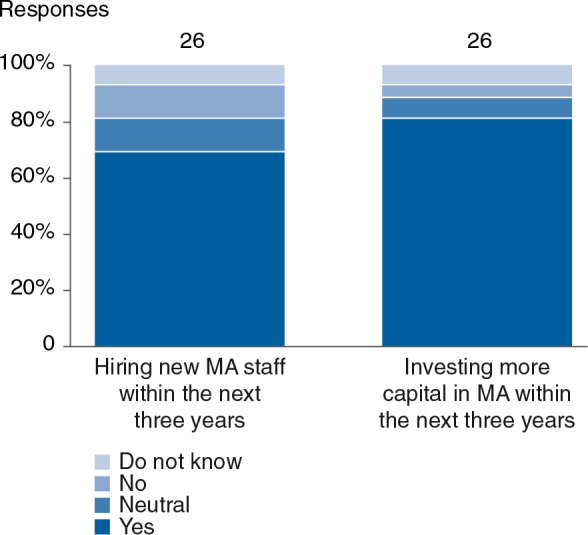
Seventy percent of respondents indicated a need for new MA staff in the next 3 years. Eighty percent will invest more capital in MA.

MA activity models for different organizational entities appear intuitive (see Fig. 9): reimbursement and dossier writing activities account for the biggest chunk at the country affiliate level (>40%), whereas evidence generation is more centrally driven. Country affiliates and regional headquarters, however, will invest more in local/regional evidence generation in the future (see Fig. 10). Payer engagement will be emphasized much more, especially at the country affiliate and regional headquarter levels. This point links back to the implementation gap relating to more extensive partnerships with payers (B8) and indicates that industry has recognized this issue and is already working on closing this gap. Expanding MA activity models will require additional resources. As a result, 70% of respondents indicate they will hire additional staff and 80% plan to invest more capital in the field of MA (see Fig. 11).

## Discussion and conclusions

A key observation of this study is that implementation levels for MA excellence prerequisites are generally lagging behind their perceived importance. This observation supports the notion that MA is a comparatively young field that has only started to take off in the new millennium, as organizations face an increasingly fast-paced and complex environment. MA is still at an early stage of the learning curve – compared with more mature disciplines such as marketing and regulatory affairs – where internal company governance, processes, and tools have already undergone several iterative cycles. The regulatory field in particular has significantly evolved post-thalidomide. Instruments and processes have been developed to transpose uncertainty into controllable risks, up to the point where regulators are willing to assume certain risks with a ‘risk management attitude’ in order to grant patients timely access to therapeutic innovations, for example with the accelerated approval of certain oncology or orphan drugs on the basis of limited clinical evidence (16). Contrary to this state, in the payer and MA world, there is still a debate about addressing uncertainty in conjunction with risk aversion, resulting in a systematic ‘cost containment attitude’ of payers preventing or delaying patient access to new medicines (16). Risk management instruments to address payer uncertainty including their rational use, for example, P4P or coverage with evidence development, are still largely in the ‘experimental stage’. This will require further co-evolution of processes (including early dialogues), tools, and capabilities of payers and the life sciences industry to ensure effective patient access to medicines, clarity, stability, and predictability.

In the following sections, the author illustrates the MALEI benchmarking approach and discusses the key implementation gaps.

### Measuring ‘fever curves’

**Fig. 12.**
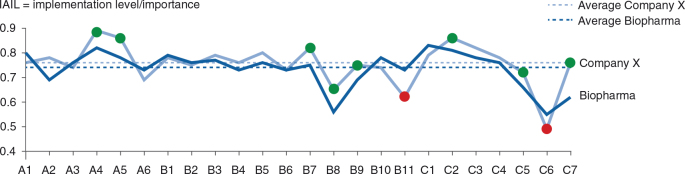
The MALEI benchmark for the biopharmaceutical industry and the profile of the hypothetical company X depicted as IAILs by excellence item (*n*(Biopharma)=47–53).

With the MALEI approach, for the first time to the best of the author's knowledge, a benchmark of MA excellence across the product life cycle has been made publicly available. Benchmarking in the field of MA may help biopharmaceutical companies in at least two ways: 1) to identify the key gaps that need to be closed to avoid competitive disadvantages, i.e., the ‘homework’ that needs to get done, and 2) to evolve a MA profile that clearly differentiates the company from the competitive field, i.e., to make strategic choices. Fig. 12 displays the ‘fever curves’ of the industry benchmark (all biopharmaceutical companies) and of a hypothetical company X. Curves depict IAILs normalizing the degree of implementation for importance. Such a single dimensional representation of the MALEI profiles allows for easy ‘signal detection’. In this example, company X is in the fortunate situation of being generally ahead of the curve. Particular strengths (vs. industry benchmark) encompass executional excellence (plan alignment – A4, A5), payer engagement, and focus on outcomes (alternative reimbursement models – B7, payer partnerships – B8, services ‘beyond the pill’ – B9), as well as the strategic leading role of MA (C2). Building further on these strengths will certainly make company X an innovation leader in MA. Company X may be advised to perform a deep dive into their policy capability (B11). Closing the gap here would ensure alignment with the overall strategic direction this company has decided to take, that is, to be proactive and coshape the external environment rather than to take a reactive approach. Although comparably strong in some aspects of MA talent management, that is, the systematic development of suitable company-internal talent into MA access roles (C5) and the rotation of MA talent through roles outside of MA (C7), company X may want to evaluate whether it would not be beneficial to more systematically rotate emerging marketing and general management talent through MA roles (C6). This step would certainly help company X in accelerating its journey towards MA innovation leadership.

### Embedding the MA perspective as early as possible

Fueling future growth through investments in R&D is essential for research-based pharmaceutical companies. On the other hand, however, healthcare budgets across the world are more and more tightly controlled. Therefore, technologies in development need to be evaluated to see whether they maximize the return on investment and the societal impact of R&D (17). This situation calls for a value-oriented approach to R&D and implies that a reimbursement and MA perspective should be at the core. Therefore, applying health technology assessment (HTA)–type methods early in R&D will not only help indicate the probability of return on investment but also provide guidance for further value-oriented clinical development and the formulation of the value story. *Early HTA*, as this approach is called, is an emerging field (17). As such, it is no surprise that early involvement of MA in R&D (i.e., already in target selection and preclinical validation – A2) was rated low in terms of degree of implementation in the MALEI survey. Early HTA and health economics considerations are certainly only one aspect of complex R&D decision support. They need to be embedded into the overall company R&D portfolio strategy and linked to clinical considerations. Close integration across functions involving multiple stakeholders is paramount. The ultimate goals are either early and therefore cheap failure or effective profiling of candidates in terms of economic and clinical value. Advancing early HTA should therefore be a key consideration of research-based pharmaceutical companies. For an overview of methods, applications, and limitations in the early assessment of medical technologies, the reader is referred to the comprehensive review article by Ijzerman and Steuten (17).

### Collectively shaping the new reality and regaining payers’ trust

Payment and incentivization based on health outcomes are the key elements of a new healthcare reimbursement reality. Payers across the world are experimenting with alternative provider and pharmaceutical reimbursement models. The ACA in the United States and the resulting ACOs, incentivized on outcomes, are likely to change the business in the United States, but are as yet in an early stage. ACOs are groups of providers aiming to provide integrated and coordinated care to Medicare patients driven by outcomes and sharing any achieved savings (18). The classical pharmaceutical model of ‘pushing products’ will not work here. Instead, a partnership model with common goals will be paramount. Common goals that meet the interest of both ACOs and manufacturers to build partnerships could be for example adherence improvement and disease management programs. Payers as partners – helping them deliver the best possible care for their patients efficiently – could be understood and implemented as a joint task, but will require a paradigm shift for biopharmaceutical companies: from product to solution business and health outcomes delivery. There are in the meantime numerous examples for pharma–payer partnerships (PPPs) that go beyond ‘classical’ cost-sharing and performance-based risk-sharing arrangements (19) towards value-added PPPs to improve patient outcomes. Examples from Germany include the setup and management of integrated care networks, for example the schizophrenia care project of AOK Niedersachsen in collaboration with Janssen-Cilag through its subsidiary I3G (20), or joint health services research such as the Initiative Dementia Care conceived by AOK Bayern and the AOK Bundesverband with Pfizer and Eisei (21). In the United States, several real world evidence (RWE)–led PPPs were forged, such as the partnership between AstraZeneca and WellPoint/HealthCore (22) or more recently between Lilly and Humana (23). These partnerships are about sharing expertise in order to develop ways of integrating data from multiple sources to allow for evidence development, feeding back real life information into the drug discovery process, and identifying ideas to improve outcomes and lower costs. A recent survey of US and European payers as well as representatives from the pharmaceutical industry revealed the major obstacles but also showed viable ways forward for successful partnership relationships (see Table 1) (9). The trust deficit between biopharmaceutical companies and payers has been identified as one of the biggest issues. With emerging value-added and RWE-led PPPs, a window of opportunity has opened for biopharmaceutical companies to regain trust with payers and to jointly shape the new reality.

**Table:** *Table 1*. Aligning objectives and building trust on both sides will be key for pharma–payer partnerships

	Pharmaceutical companies	Payers

Major obstacles for a partnership relationship	• Pharmaceutical companies are not trusted as it is believed they are biased towards their own products rather than those of competitors • Approach payers ad hoc and randomly versus have a systematic and comprehensive strategy • Use services defensively to push their own products • Prefer placebo-controlled over comparative clinical trial data (head-to-head) and RWE for value demonstration • Publish study findings selectively versus comprehensively (positive and negative)	• See medication costs as the biggest problem (88% of surveyed payers versus 44% of pharmaceutical company representatives) • Prefer cost containment (drug and non-drug) over outcomes-based approaches • Use stricter coverage criteria from the outset versus cost- or risk-sharing agreements with manufacturers • Struggle with tactical challenges i.e., implementation of healthcare reforms and modernization of IT/infrastructure
Way forward	• Respect payers’ role in managing care • Restore trust and increase transparency • Help payers and healthcare providers address their biggest challenges – Help payers design scalable and replicable outcomes-based approaches through advanced data integration and analysis – Develop programs aimed at better adherence – Advance risk-sharing towards global payment models and disease management	• Be open to or initiate innovative solutions for health outcomes delivery jointly with manufacturers – shape the future of healthcare collaboratively • Take a balanced position towards drug cost as the single most important cost driver in healthcare to manage • Acknowledge the value of pharmaceutical research in advancing healthcare and wellbeing

Results of a survey of 30 US payers, 30 European payers, and 18 pharmaceutical company representatives (9).

### Advancing MA human resource and talent management

Talent in MA is much sought after and will be even more so, not only to handle the surge of launches in the near future but also to further expand MA activities and capabilities, as the results of the MALEI survey show. Some companies have started to experiment with innovative approaches to fill their MA talent pipeline by systematically developing and redeploying internal resources to MA functions (see MALEI item C5: ‘suitable company-internal talent should be systematically developed into MA roles’). This is triggered by strategic choices to accelerate MA capability building but also in response to strained job markets, especially in emerging economic regions. Such systematic programs usually include a rigorous personnel selection process and on-the-job training, as well as a concomitant academic formation in MA to provide the future MA managers with the necessary theoretical understanding of MA concepts. Furthermore, these programs try to foster a ‘MA academy’ and team spirit through MA trainee joint project work. Apart from securing MA talent as a basis, biopharmaceutical companies should be concerned with spreading a MA mindset throughout their organizations. This was shown in a study published in 2013 that recommended that high potentials rotate between commercial functions and MA roles prior to taking on general management positions. It also suggested that high potentials in MA should be encouraged to take on roles outside their functions (11). Both proposals, rated as comparatively underimplemented in the MALEI survey (C6, C7), strengthen mutual understanding and help develop MA expertise. Moreover, for MA experts, such an approach would help rebalance functional expertise (as expressed in years of MA experience) versus understanding other aspects of the business versus other competencies such as strategic thinking, the ability to drive results, collaboration, and influencing skills for further professional growth (11).

In conclusion, MA (launch) excellence needs to be further developed in order to close implementation gaps across the entire product life cycle. As a comparatively young pharmaceutical discipline in a complex and dynamic environment, this endeavor will require strategic focus and dedication. The MALEI benchmarking tool may help guide decision makers to prioritize their endeavors.

## Conflict of interest and funding

The author has not received any funding or benefits from industry or elsewhere to conduct this study.
